# At the interface between the formal and informal, the actual and the real: a realist study protocol for governance and accountability practices in urban settings focusing on adolescent sexual and reproductive health and rights

**DOI:** 10.1186/s12939-022-01644-1

**Published:** 2022-03-23

**Authors:** Sara Van Belle

**Affiliations:** Institute of Tropical Medicine, Antwerp, Belgium

**Keywords:** Accountability, Local governance, Sexual and reproductive health and rights, Urban health, Local health system, Realist evaluation

## Abstract

**Background:**

This paper presents the protocol of a study that aims at exploring how different multi-level governance arrangements impact on sexual and reproductive health of adolescents living in informal settlements. The overall objective of this study is to contribute to a better understanding of the causal chains underlying accountability in sexual and reproductive health for adolescent girls and young women living in urban informal settlements in low-and middle-income countries.

**Methods:**

The overarching methodology is realist evaluation. The study adopts a case study design, through which governance and accountability practices in Mumbai, Delhi, Cotonou and Kampala will be examined. Different social science methods to develop and test a programme theory will be used. Heuristic tools for the analysis of the accountability ecosystem and mapping of governance arrangements, drawing from contextual political analysis and critical realism, will be developed in order to identify the intervention-context-actor-mechanism-outcome configurations.

**Discussion:**

The methodological approach is geared towards building robust case-based explanation with due attention to context and the roles of different actors. The combination of different social science methods will lead us to a better grasp of the inherently political nature of social accountability.

## Background

Two-thirds of the world’s population will live in urban settings by 2050. Nearly 90% of 2.5 billion ‘new’ urban residents will live in Africa and Asia. Sixty % of the global urban population will be under 18 by 2030 [1]. A considerable proportion of these urban adolescents live in insecure environments, exposing them to a number of sexual and reproductive health-related risks. Through overcrowding, hyper-mobility, entrenched poverty, an informal economy based on middlemen and bad infrastructure, informal urban settlements expose their vulnerable residents, including adolescent girls and young women, to health risks. Health system challenges include denial of access to a quality public health system. When services are available, residents of informal settlements often face poor quality of care, stigma, discrimination by providers, and lack of recognition of citizenship (or non-portability of health entitlements in the case of migrants). Unregulated private providers often overcharge users for care of poor quality. The privatization of public goods (such as hygiene facilities), the absence of specific adolescent sexual and reproductive health and rights (ASRHR) and sexual violence services, and delays in referral of emergencies due to traffic congestion present additional challenges for adolescent girls and women. Yet, the urban environment, including informal settlements, can be a site for adolescents’ empowerment, specifically of adolescent girls: it can provide opportunities for innovation through multi-actor engagement, self-organisation and emergent collective action, linking urban city authorities, NGOs and grassroots or community-based organisations. Cities are arenas where actors engage in consensus-building strategies but also in resistance and protest, to enforce accountability towards vulnerable groups.

There is a dearth of evidence regarding the macro- and meso-level context conditions that increase the vulnerabilities of adolescent girls in the informal settlements of cities and which lead to poor adolescent sexual and reproductive health and rights (ASRHR) outcomes. Research into the relationship between the physical environment, the (un)safety of public space in informal settlements and adolescents’ needs for protected space and privacy is a priority.

In this study, I focus on the sexual and reproductive health and rights of adolescent girls and young women living in informal settlements, and more specifically on life skills education, sexual health, sexually transmitted infections, sexual violence and rights to health. At the core is the examination of the causal chain between accountability practices, sanitation and sexual and reproductive health service delivery in informal settlements, and health outcomes of adolescent girls and young women in Kampala (Uganda), Cotonou (Benin), and New Delhi and Mumbai (India). I will look specifically into the intersections between gender, citizenship, poverty, ethnicity, caste and religion.

### Adolescent girls in informal settlements: health, precarity and accountability

Since 2007, more than half of the world’s population lives in cities. Urbanization is only expected to increase during the next decades, with expansion or consolidation of informal settlements in many low- and middle-income countries (LMIC). Within informal settlements, adolescent girls are a vulnerable group. Besides the burden of disease related to the socio-economic and environmental conditions (overcrowding, extreme poverty, pollution and social exclusion related to caste, ethnicity or being a migrant), they experience specific disadvantages and risks related to their sexual and reproductive health (SRH) and rights, and prevailing gender norms. Levels of sexual violence in slums are high. Among 313 surveyed young adult women in Kampala, 32% said to have been raped [2]. Violence towards adolescent girls is associated with obtaining resources such as food and money, and the absence of trusted family members. In a cross-sectional study in Kampala, 73% of adolescent respondents reported that it was “*common for strangers (but also relatives) to force young females to have sexual intercourse without consent*”, likely resulting in unintended pregnancy [3]. Pommells et al*.* demonstrated the link between access to sanitation facilities and sexual violence [4]. The lack of access to sanitation facilities (‘sanitation insecurity’ [5]) does not only expose them to a range of infectious diseases, but also to multiple stressors, inadequate menstrual hygiene, sexual violence and adverse pregnancy outcomes [6, 7]. In India, not having a house toilet doubles the risk of sexual violence [8]. High levels of child marriage and childhood pregnancy contribute to the health risks. Adolescent girls often prefer living in groups in overcrowded rooms over living with family in rural areas, as a way to escape the lack of voice and the overburden of household work [9]. Others live with their family, but are married off and become pregnant at a precocious age with increased risks of birth complications. In Uganda, 40% of adolescent girls is married by the age of 18 and in in India and Benin, early marriage is not declining, despite it being illegal [10, 11].

A systemic analysis of upstream political determinants of health service delivery for vulnerable groups is neglected in current health research. Informal settlements are often illegal, which means that local authorities do not recognise their inhabitants as citizens with legal entitlements. Thus, their right to access to public services, including health services, waste management and sanitation, is ignored. Delhi has 750 slum clusters. The Indian government only recognises two types of slums: legally recognised or “notified” slums and illegal informal settlements [12]. People living in notified slums experience less deprivation because of better access to public services [13]. In Kampala, slums take up one quarter of the total city area, housing 60% of the population. Forced eviction has led to mistrust of the municipal authorities. The latter relies on NGOs to provide adequate sanitation [14].

The fact that circumstances are dire for many does not mean that residents are passive nor that they powerless [15]. If effectively supported, adolescent girls can emerge as a powerful force for change and agency. In informal settlements, they are often part of informal networks of grassroot organisations, community leaders and community-based organisations. Informal leaders act as power brokers and link up to formal actors, such as local authorities, politicians and (international) NGOs, to obtain public goods for the slum communities. Such de facto governance arrangements have been labelled ‘webs of informal governance’ [9], ‘hybrid’ [16] or ‘real’ governance [17]. These authors refer to the emergence of specific accountability relationships in marginalized communities, which are best considered as accountability ecosystems: “*complex systems of informal and formal accountability arrangements involving multiple actors with a wide range of roles, responsibilities and interactions across levels, from the transnational to the local level*” [18]. More recently, such accountability practices are described as ‘organic’ [19].

### Accountability interventions in health in low- and middle-income countries

In the last ten years, accountability has risen high on the global health agenda, where it has been interpreted as holding those in power to account. This began with the UN Secretary General’s Every Woman Every Child Campaign that put the spotlight on accountability as a way to improve maternal, neonatal and child health and address the disappointing results related to MDG 5 (the reduction of maternal mortality and access to reproductive health). However, the concept is quite nebulous and its application in global health is influenced by specific disciplinary perspectives and trends, including New Public Management, human rights and ethics [20].

In recent years, social accountability interventions have proliferated in LMIC [21, 22]. To influence the accountability ecosystem, two main routes have been followed [18]. First, civil society organisations have been demanding accountability on behalf of vulnerable, underserved groups (such as adolescent girls and young women, but also other vulnerable groups) through social mobilization, protest and public interest litigation. These oppositional strategies can be contrasted with consensus-oriented approaches that are primarily driven from within the health system and seek to establish a better dialogue between the communities and upstream (local government or state government) or downstream (district health managers, health service providers) actors in the health system. Participatory audit processes or citizen participation structures (eg. village health committees) are used to seek redress for wrongdoing in a collaborative manner. For instance, NGOs working on accountability in SRH in Uganda prefer consensus-oriented strategies, referring to reduced civic space and governmental repression and the influence of external donors’ preferences, although NGOs have used strategic litigation to address maternal mortality [23, 24]. In India, a mix of oppositional (eg. public interest litigation) and consensus-oriented strategies are being used to hold government and increasingly, private sector actors accountable [24–26]. Benin has a high teenage pregnancy rate and a high maternal mortality ratio. Interventions therefore focus on, by way of NGOs, girls’ empowerment to strengthen their voice on the one hand, and, on the other hand, supply-side interventions, such as the improvement of the quality of care through maternal audits. Recently, also, a more progressive abortion law was adopted in Benin, for which NGOs advocated [27–29].

Both oppositional and consensus-oriented strategies of accountability share the underlying assumption that better accountability would improve health system governance and thus lead to improved quality of care and health outcomes [30]. However, they are grounded in competing Western political theories (Mouffe’s agonistics [31] and Habermas’ consensual deliberation [32] respectively). The causal chains of these accountability strategies have rarely been empirically explored and compared in urban informal settlements in LMIC. Moreover, it is not clear which strategy works best in which political context, at which stage of the policy process and how (in terms of processes, underlying mechanisms and outcomes).

### Research objectives

The overall objective of this study is to contribute to a better understanding of the causal chains underlying accountability in sexual and reproductive health for adolescent girls and young women living in urban informal settlements in LMIC. In line with the steps of the realist research cycle, which I discuss in the next section, I define the specific objectives as follows:

1. To develop the initial programme theory on accountability in sexual and reproductive health towards adolescent girls and young women living in informal settlements
2. To empirically test and refine the initial programme theory in four settings
3. To refine the programme theory that specifies the mechanisms and context conditions driving accountability towards adolescent girls and young women in informal settlements
4. To develop methodological guidance regarding realist research for complex problems with specific attention to the impact of local (political) context on intervention strategies

This study will contribute to the knowledge management strategies of the World Health Organization’s (WHO) Community of Practice on Accountability in Reproductive Health and the COPASAH global network, the community of practitioners on accountability and social action in health. It will connect endeavours of decontextualizing, synthesising and disseminating knowledge from local practitioners and local collective action to policy-making levels and practitioners in other settings.

## Methods

I will adopt realist evaluation (RE), a research methodology grounded in scientific realism. Originally developed by Pawson and Tilley [33], it aims at answering not only the effectiveness question ‘does it work?’, but also the causal questions ‘how, why, in which conditions and for whom’. Realist evaluation is an approach that fits complex topics well [34]. It combines a realist ontology with a weak constructivist epistemology. Focusing on mechanistic causal explanation, RE considers that outcomes or events result from generative or emergent causal mechanisms that are triggered in certain contexts. Realist evaluation provides not only a methodology to develop, test and refine theory, but also to bolster comparative case study design.

**Fig. 1:**
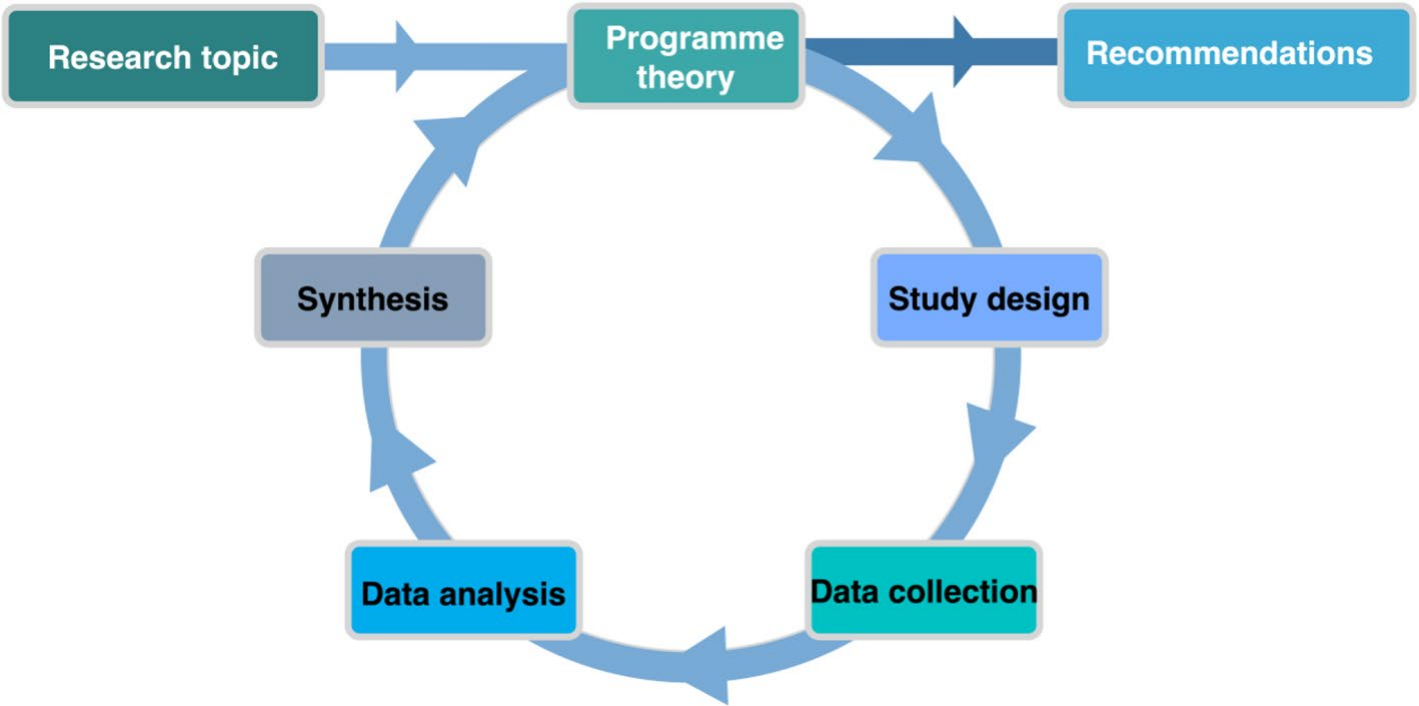
The realist theory-building cycle, adapted from Marchal et al. [37]

RE studies start and end with theories. Its research cycle is aimed at theory-building (Fig. 1). So-called programme theories (PT) spell out how outcomes are expected to be attained, in which conditions (context) and how (mechanisms). These programme theories are to be refined through repeated empirical testing. The ultimate aim is to build a theory of the middle range on a particular social phenomenon. The middle range theory, a notion developed by Robert K. Merton, lies between macro-level ‘grand’ theories and context-specific narratives [35, 36].

In any explanation of social and political determinants of health inequities, context matters. Any social phenomenon to be empirically observed and subsequently explained by means of political and social determinants is set in a specific loco-temporal (place/time) context and any action is to be situated at a particular level or scale [38]. These context conditions are often only in part open to empirical observation, as they occurred prior to the actual data collection in case of retrospective research. Some of these context features and their impact can only be understood if their processes are traced back to events and processes in the past, and thus, outside immediate empirical observation [39, 40]. For example, if one wants to explain the social exclusion of current Muslim youth in urban India, one needs to go back to historical events and processes of formation of citizenship and state-Muslim community relations. Similarly, women’s protest for better care can only be adequately understood within the context of the emergence of the women’s rights movement in India in the 1970s. Social determinants research rarely focuses on these processes of social change and transformation, and if not explained, such studies can appear deterministic [40, 41].

In contrast with ‘traditional’ variable-based successionist causal explanation, dominant in research on determinants of health inequities, RE offers a case-based approach to examine causal relationships, exposing the generative mechanisms that explain how in specific conditions an intervention brings about an outcome (or not) [42]. By means of its emphasis on causal pathways or chains, in which actors’ agency and social structures interact to produce outcomes, RE is well-equipped to explore the intersecting vulnerabilities and the impact of context in this study. Intersecting vulnerabilities can indeed only be partially uncovered by the variable-based explanation in health determinants research [43].

My analytical strategy is inspired by the work of critical realist Margaret Archer. One of the key theoretical premises of realist research is that it analyses a stratified social reality, which consists of three layers: the empirical, which is observable and can be studied, the actual and the real [44]. In contrast to constructivist approaches, it distinguishes what is knowable (epistemology) from what really exists (ontology). Realism positions itself between positivism and constructivism. It has in common with constructivism that we can only gain partial knowledge of social phenomena (the empirical) from our own perspective or vantage point. Our position in the social world determines how we derive knowledge from it. Hence, we need to use a range of methodologies and tools from different disciplines to build theories on social phenomena and understand what is happening (ie. ‘the actual’), which is a reality outside of how we perceive people’s actual practices. To understand why a certain social phenomenon in a given context emerges, we need to refer to the ‘real’ layer. The *‘real’* is the domain where the interplay between social structures and human agency leads to causal mechanisms or powers generating the social practices in the actual realm [45].

To capture social practices in a given context and their effect on change, Archer developed an analytical framework [45] that fits our study approach well. The Structure-Culture-Agency (SAC) nexus is the core tenet of Archer’s frame, where “*full significance is accorded to the timescale through which structure, culture and agency themselves emerge, intertwine and redefine one another, since this is the bedrock of the explanatory format employed in accounting for any substantive change in social form.*” Archer uses a temporal analytical sequence to analyze the emergence of social change. In my study, I consider accountability as inherently relational and dynamic, with the potential to transform social reality or to perpetuate the status quo [46]. The interaction between social structure and agency is central to understanding what drives actors’ accountability practices. Formal governance arrangements as well as informal norms (Time 1—T1) are indicative of structure and culture and predate emergent accountability practices that are grounded in actors’ agency and collective agency (T2-T3) and are potentially transformative. T4 is the phase when social change occurs, improving responsiveness towards adolescent SRH needs in informal settlements, or not, when the governance system reverts to the status quo. Following Archer’s analytical framework, governance can be considered as embedded in structure and culture, predating the accountability practices we empirically observe. We thus need to apply a historical lens to the analysis of governance relations and accountability practices. These do not happen in a temporal vacuum: relationships and practices are dynamic and co-evolve with changes in context. For example, a grassroot organisation’s accountability strategy may have evolved from protest actions towards seeking consensus and collaboration with local authorities, dependent on the broader state-citizen context, the relationship with local authorities, changes in local political power and professionalization of the organisation or changes in the leadership of the organisation itself.

In realist research, context matters a lot. In applied research domains, such as health, outbreak control, education and implementation science overall, we are experiencing the “contextual” turn, a shift from context-aware to context-driven research that pays more attention to local contingencies. [47–50]. However, health researchers seem to have been grappling with ways to define context, and to use methods that go beyond the mere enumeration of political, social or cultural context factors [51]. Some research articles analysing contextual drivers or ‘macro-level factors’ in relation to tuberculosis and diabetes are a case in point [52–54]. Many, if not most such studies struggle to explain exactly why and how ‘context’ impacts on the study outcomes or results.

Health inequities research has focused on exposing the determinants of inequity, following the influential 2005 WHO Commission on the Social Determinants of Health**.** The focus on determinants, drivers or root causes remains highly influential and has been transposed to research zooming in on political determinants and drivers within the context of the Sustainable Development Goals and of reaching Universal Health Coverage [55, 56]. Also in this field, research that focuses on determinants, macro-factors or context drivers has been criticized for giving way to overtly deterministic thinking and not going much beyond stating that context conditions matter [51]. Such studies often present correlations between a context condition and a disease – for example the correlation between countries in conflict and leishmaniasis [57]. From a realist perspective, constant conjunction is no substitute for causation. Furthermore, such studies frequently do not provide the means to adjudicate why a certain context condition matters more than another, nor do they consider the complex interplay between structural conditions and agency. Even if the political context is mentioned as a structural determinant—collective action and agency, for example in the form of actions performed by civil society, grassroots- or community-based organisations, are often left out of the explanatory equation [45].

Next to the temporal dimension, one of the important dimensions of ‘context’ is evidently the geographical location, with ‘place’ often conveying a sense of meaning (including negative meanings) [58]. ‘Slums’, or, informal settlements as a study context illustrate this very well. For a long time, the term ‘slum’ was indicative of a negative urban planning discourse, with informal settlements considered solely in a pejorative sense as a ‘negative space’ in terms of governance and urban planning. In such discourse, there was nothing else one could do but to eradicate slums. Slum eradication, however, has nowadays, at least on paper, been replaced by a discourse of slum upgrading and urban informality and creativity [59–61]. Moreover, with the Covid-19 pandemic, authors have noted that the relative absence of local authorities in informal settlements or poor marginalised city areas does not mean that there is no health governance, as people tend to self-organise to access health services, installing handwashing stations or getting supplies delivered in the local hospital in the favela in Rio de Janeiro [62]. Governance in informal settlements does not automatically equate with a negative or empty space. To the contrary, it acts as an essential corollary to the ‘formal’ sphere: it is the informal governance relationships on which the formal world depends, and there are fluid boundaries between both spheres [63].

Below, I present the methods in relation to each step of the realist research cycle (Fig. 1), which starts and ends with theory [37]. Initial programme theories are refined through a process of accumulation of insights and evidence and specification of the findings. The end result is a programme theory which tells us how in certain context conditions, actors’ strategies might work or not work and why. I refer the reader to [64] for a succinct overview of the main principles and definition of terms used in RE. It should be noted that while I developed the study in response to a call for individual post-doctoral fellowships of the Flemish Fund for Scientific research (FWO), in each study country, one main researcher will lead a team of 2 to 3 junior researchers. They will be involved in data collection, data analysis and writing up of the findings.

### Formulating the initial programme theory

The initial programme theory that is the starting point of this study builds upon previous research on accountability towards vulnerable groups in an urban and a rural local health system in Ghana [65]. That study concluded that public accountability towards the most vulnerable groups in urban and rural health systems needs to be enforced by the groups themselves through voice and collective action. Table 1 presents the narrative version of the resulting programme theory.

**Table 1:** The programme theory of the Ghana study [66]

• Public accountability is actualised when actors are answerable to the public and remedial action is undertaken
• Public accountability requires both answerability and enforceability. The capability of the District Health Management team, the sexual and reproductive health NGOs and of partnerships and to inform, evaluate and report in an open manner is grounded in compliance and persuasion. Persuasion is founded on trust and reciprocity, grounded in actors’ perceptions of shared values and norms, goals and of future gain
• Enforceability is grounded in the capability of the public to demand accountability on the one hand, and in meta-governance, i.e. the function exercised by state actor(s) of regulating, supervising and sanctioning, on the public’s behalf, on the other hand. Enforceability is required to ensure answerability, especially to the public
• The accountability practices of health actors are embedded in vertical, horizontal and partnership governance arrangements, which are insufficient in themselves to ensure public accountability. Accountability practices in vertical governance arrangements are strong, if they are grounded in effective hierarchical power. The number of actors in the local health system, the social fabric and the local power structure are important local context conditions that influence the relative strength of public accountability practices
• Public accountability requires a public demanding accountability and/or a meta-governor that monitors and enforces when necessary the actors to be accountable. The meta-governor requires adequate resources, decision space and the capacity to play its role effectively
• Public accountability practices operate along four dimensions of accountability (social, political, organisational and provider dimensions). To be publicly accountable, each actor needs to put in place a specific configuration of public accountability practices, based on the public it claims to serve, its organisational profile and culture, and the governance arrangements in which it is engaged. Multi-level governance arrangements weaken public accountability when there is confusion over roles and responsibilities between actors

In practice, the above programme theory will be refined on the basis of a review of accountability in sexual and reproductive health and rights [18], an ongoing realist review on adolescent digital empowerment strategies and accountability, and a review of the literature on access to adolescent-friendly services in informal settlements in LMIC.

### The study design

Realist research is method-neutral, and the study design needs to enable testing of the initial programme theory. Understanding the context conditions for improved accountability towards adolescents living in poor urban neighbourhoods or informal settlements requires an in-depth understanding of the mechanisms underlying the observed practices and agency. A causal case study design allows for the study of social interaction and actors’ practices and the analysis of the underlying causal mechanisms and context conditions [67]. Causal case study methods have emerged in comparative politics, which has grown into a full sub-discipline [68–70].

In this study, the case is defined as accountability practices to enhance vulnerable adolescents’ sexual and reproductive health and overall well-being and empowerment. The study sites are informal settlements in Kampala (Uganda), Cotonou (Benin), and New Delhi and Mumbai (India), and more specifically urban poor neighbourhoods, where marginalised communities or groups reside. A layer of context that may be relevant at the meso-level is local urban governance, local political context and the history of grassroots organisations working with adolescents in urban poor neighbourhoods. In India, I included two different mega-cities, New Delhi and Mumbai, where different political parties are in power at the city- and state-level. Elements of the macro-political context, including the political system, and issues of democratic space, social inclusion and citizenship, will be captured through the differentiation between countries.

In each city, a grassroot organisation is selected as an entry point for fieldwork. These organisations work with and for adolescent girls and young women with intersecting vulnerabilities, such as belonging to a religious minority, gender, caste or poverty. They are active in poor urban neighbourhoods or informal settlements where these vulnerable groups reside.

This overall set-up represents different layers of context conditions, at the micro- (adolescents’ intersecting vulnerabilities), meso- (grassroot organisation) and macro- (political) level, as we assume that these play a role in accountability strategies and practices.

### Data collection

Realist evaluation is method-neutral: any data collection method that yields data required to test the initial programme theory can be used.

#### The governance ecosystem

The governance ecosystem can be defined as the governance actors and the relationships that inform the actual governance arrangements and practices as they are operating in informal settlements. I will map both the formal and informal governance spheres.

To map the formal governance sphere, we will collect data through in-depth interviews with city- and local (health) authorities and representatives of local community-based organisations. We will also conduct a review of policy documents, with the aim of analysing relevant policy documents originating at different levels relevant to adolescent health and well-being, including the municipality and district / sub-district levels.

To map the informal governance sphere, we will use data collected through interviews with representatives of grassroot or community-based organisations, community or religious leaders, and informal middlemen. The latter are known to negotiate access to public services on behalf of vulnerable groups. We will also include local politicians, municipal administrators responsible for youth and / or adolescent health, youth leaders and staff of civil society organisations working with adolescents and young adults.

Finally, we will deepen the analysis by focusing on one grassroot organisation in each study site. We will map the governance relationships of the organisation, its (historical) relationships with stakeholders, and its context, including the informal settlement, the municipality, and the central and decentralized policy-making levels. To this end, we will carry out additional in-depth interviews and review the organisation’s documents and records.

Interview guides will be prepared for the interviews and the focus group discussions. In line with realist principles, the topics will be based on the initial programme theory, whereby for the adolescent girls, the focus will be on their experiences, their living conditions and their views on their health and rights. For the other respondents, the questions will probe for their views on and experience with accountability, their agency and their context, including the governance arrangements.

#### The accountability ecosystem

We will map the actual accountability practices, again using the grassroots organisation as entry point. The focus will be on self-organisation and emergent collective action to demand services through agonistic and/or consensus-building strategies [31, 32]. These strategies are based on different causal pathways and underlying mechanisms, such as trust and reciprocity, but also voice or neighbourhood solidarity. I will apply an adapted accountability mapping tool I developed in Ghana [71]. Local researchers will use in-depth interviews and focus group discussions to collect data on local accountability practices. The respondents will include the adolescents engaged in grassroots organisations, the leaders and volunteers of the organisations and other networks in the neighbourhood, community and religious leaders, and middlemen or women who negotiate access to services for vulnerable groups. [9, 66]. I will select adolescents who are willing to be interviewed from the focus group discussions.

### Data analysis

In realist research, the context-mechanism-outcome configuration is used as the main heuristic for data analysis [33]. Given the multiple sources of potential methodological confusion [37], refining methods for realist analysis remains a priority.

In practice, we will analyse governance arrangements and accountability practices in 2 steps. First, we will qualify the formal and informal governance relationships with actionable governance terms such as ‘reports to’, ‘is formally accountable to’, ‘is informally accountable to’, ‘informs’, ‘monitors’, ‘supervises’, ‘funds’, etc. This method was piloted in the previous study on accountability in the urban health system in Ghana [65] and will be adapted considering qualifiers of the WHO Euro governance mapping tool [72]. Second, we will analyse and appraise the accountability practices and strategies (the accountability ecosystem), which includes analysing the formal mandates of actors and the intersectoral action related to adolescent health, answering the question: who is supposed to be accountable to whom? To analyse and appraise accountability practices, we will assess the actual practices in four dimensions: the social, political, organisational and service provision dimension. The results will be presented in spidergrams that present accountability according to these dimensions [65, 73]. A separate analytical tool will be designed to differentiate accountability practices in agonistic and consensus building strategies, and to assess the potential for transformative agency.

A context mapping tool will be designed to extract and analyse relevant context data from the in-depth interviews and focus group discussions. This tool will include temporal dimensions such as the evolution of the local governance context (eg. change in political power at city level; growth of grassroots organization) and place-based dimensions (including meaning / perceptions of different actors regarding the neighbourhood). The output of these steps will be thick descriptions of the case in each study site.

Based on the governance and accountability mapping and analysis, we will develop the most plausible explanations of the observed outcomes of accountability towards vulnerable girls and female adolescents in each site. In practice, this analysis starts with applying an adapted version of the heuristic tool commonly used in RE, which we call the ICAMO configuration (for Intervention-Context-Actors-Mechanism-Outcome), to the thick case descriptions. We use the ICAMO configuration instead of the CMO, as it stimulates the researcher to focus the analysis of the causal pathways on the actors. In this case, adolescents living in informal settlements should not be a priori considered to be a homogeneous group. Adding ‘Intervention’ to the heuristic helps in describing the actual interventions, policies, programmes, activities, etc. that shape the outcomes (or not) [74].

It is at this stage in realist research that the mechanisms are identified: mechanisms are the drivers of actors’ actions, generated under specific context conditions. In this study, they may include trust, social exchange, empowerment, voice, etc. For each site, I will identify and describe the salient context features that may influence how individual and collective action for accountability generates social change in the system or not.

The analysis results in ICAMO configurations, formulated in a narrative that includes the practices reflecting actors’ agency, and ‘if …, then …, because …’ clauses. We will use process-tracing techniques, developed in political science, as a means to verify the robustness of the causal inferences made in the ICAMOs. These include four main tests: smoking gun, hoop, double decisive and straw in the wind [75].

### Synthesis

The last step of any realist study is the abstraction of the findings as represented in ICAMO configurations to the level of a refined programme theory. The in-case synthesis consists of comparing the ICAMO configuration(s) with the initial PT, whereby the latter is adapted: some clauses may be confirmed and others refuted, while for other parts, there may be no evidence for confirmation nor refutation. After step 4, we will have 4 programme theories, each resulting from empirical testing in one city.

The cross-case analysis allows for a more powerful refining of the initial programme theory. According to Beach and colleagues [67], mechanism-based methodologies such as realist research can benefit from causal case study comparison, which allows testing theories on a social phenomenon through the combination of within-case and cross-case analysis. There is, however, little guidance on how to combine within-case analysis and cross-case comparison. Our study, and all studies looking at complex, multi-level structure-agency interactions meant to generate social change, reflect the methodological issue of equifinality, where the same outcome can be reached by multiple pathways given different sets of context conditions. One of the ways to analyse across cases is Mill’s methods of agreement, through which an initial programme theory is revised by eliminating conditions and we will use it to disconfirm clauses in the initial PT as being a necessary cause [67]. We will do this by comparing the city-specific PTs against the initial PT and charting the different layers of context conditions as presented by the cases.

## Discussion

Since the publication of the seminal work of Pawson and Tilley in 1997, realist research has taken hold in applied research domains, such as education and health. However, several methodological questions remain. First, in my experience, researchers applying RE in public health tend to favour individual-level, cognitive-psychological mechanisms to the detriment of meso-level (team, organisation, network) and macro-level (social system) mechanisms [20, 75]. Second, methods to explore how social structure and agency mutually bring about change are under-developed. Third, there is little guidance on assessing the interaction between context and mechanism, on how to extract the salient features of multi-layered contexts, and on how to specify the temporal context in which causal mechanisms are expected to be triggered. With this study, I argue that innovative methodological developments from political science, most notably in terms of causal case study design and methods to strengthen causal inference, can be usefully combined to address some methodological challenges of doing realist research.

One of the key powers of realist evaluation, its focus on theory-building, has been left somewhat underused in global health research [76]. Most studies do not reach the stage of refining a programme theory that can be used by researchers as a starting point for a new theory-building cycle in other settings [49]. This may be due to the fact that realist evaluation, synthesis and review have been mainly applied within the context of health programme and project evaluations, and in PhD research in the field of health in LMIC. Short-term project funding often sets limitations to the duration and depth of studies. This study builds upon my previous research, indeed taking the end point of that study as the starting point for this study and will thus offer a second round of adaptation on the basis of the case studies in India, Benin and Uganda.

Finally, in terms of content, this study will contribute to refining the theories that underlie accountability for adolescents and their sexual and reproductive health and rights because of its focus on multi-level analysis: starting from accountability practices and their outcomes, I will analyse the meso- and macro-level to assess how context and individual and collective agency interact in shaping the causal pathways underlying accountability, an all too often neglected area with accountability research.

### Ethical considerations

In this study, I recognise the vulnerability of the groups under study, in particular adolescents and young women of urban poor neighbourhoods who are engaged in the activities of the grassroots organizations [77].

I will place particular emphasis on:

- Cultural sensitivity: The data collection methods will be aligned to the socio-cultural norms and preferences of people in their daily life and within organizational settings (for example, ensuring interviews with members of the grassroots organisations do not disrupt their activities unduly). The country research teams will be composed of well-experienced researchers with a long-standing expertise in research in the local political, cultural and social context.
- Do no harm: Respondents will be well informed of their rights to withdraw from the study at any point in time and all efforts will be made to ensure that no discomfort or stress is experienced by respondents as a result of the research. This includes allowing respondents to choose their preferred location and time for interviews with care taken to ensure privacy to the greatest degree possible. Informed consent/assent, privacy and confidentiality, as well as respect for participants’ time will be prioritized. If needed, respondents who require psychological or other care will be referred to appropriate providers in consultation with the country research team members.
- Confidentiality and non-attribution: I will adopt a privacy-by-design approach when designing data collection tools. Personal data will be processed according to the EU GDPR regulations. All data is to be encrypted and password protected with the use of secured software and servers for data storage. All measures are put in place to maximize confidentiality such as pseudonymization. Once transcripts are produced, audio files are to be destroyed.
- Only the principal investigator and the country researchers will have access to raw data. All documents and audio recordings will be treated with the strictest confidence. The organizations and locations including the poor neighbourhoods and health and administrative districts where they work will be anonymized to ensure confidentiality of the respondents. All communication between the author and the country research teams will be done through an encrypted mail system. Reports will be compiled with the intention to protect the identity of respondents to the maximum while representing their views and opinions as accurately and fairly as possible.

## Conclusion

The objective of this study is to contribute to a better understanding of the causal configurations underlying accountability in sexual and reproductive health for adolescent girls and young women in urban informal settlements in LMIC. The study adopts an interdisciplinary approach towards analysing how emergent local collective action impacts on accountability in a political context with entrenched inequities. It will also show how realist research could offer a way of dialogue and collaboration across and between different scientific disciplinary communities and how its analytical strategy can be enriched with methods from political science.

## Data Availability

Data sharing is not applicable to this protocol as no datasets were generated or analysed.

## Abbreviations

ASRHR: Adolescent sexual and reproductive health and rights
CMO: Context- -Mechanism-Outcome
COPASAH: Community of Practitioners on Accountability and social Action in Health
FWO: Fonds voor Wetenschappelijk Onderzoek (fund for scientific research)
ICAMO: Intervention-Context-Actors-Mechanism-Outcome
LMIC: Low-and middle-income countries
MDG: Millennial Development Goal
NGO: Non-governmental organisation
PT: Programme theory
RE: Realist evaluation
SHR: Sexual and reproductive health
UN: United Nations
WHO: World Health Organization
